# Glutamate signalling via a MEKK1 kinase-dependent pathway induces changes in Arabidopsis root architecture

**DOI:** 10.1111/tpj.12201

**Published:** 2013-04-10

**Authors:** Brian G Forde, Sean R Cutler, Najia Zaman, Patrick J Krysan

**Affiliations:** 1 Centre for Sustainable Agriculture, Lancaster Environment Centre, Lancaster UniversityLancaster, LA1 4YQ, UK; 2 Department of Botany and Plant Sciences, Center for Plant Biology, University of CaliforniaRiverside, CA, 92521, USA; 3 Genome Center of Wisconsin and Department of Horticulture, University of Wisconsin-Madison1575 Linden Drive, Madison, WI, 53706, USA

**Keywords:** amino acids, chemical genetics, nutrient signalling, roots, signal transduction, *Arabidopsis thaliana* L., MAP kinase, *Saccharomyces cerevisiae*

## Abstract

A chemical genetic approach has been used to investigate the mechanism by which external glutamate (l–Glu) is able to trigger major changes in root architecture in *Arabidopsis thaliana* L. An initial screen of 80 agonists and antagonists of mammalian glutamate and GABA receptors, using a specially developed 96-well microphenotyping system, found none that replicated the response of the root to l–Glu or antagonized it. However, a larger screen using >1500 molecules bioactive in *Saccharomyces cerevisiae* (yeast) identified two groups that interfered with the l–Glu response. One of the antagonists, 2–(4–chloro-3-methylphenyl)-2-oxoethyl thiocyanate (CMOT), has been reported to target Ste11, an evolutionarily conserved MAP kinase kinase kinase (MAP3K) in yeast. This led to the discovery that root growth in a triple *mekk1 mekk2 mekk3* mutant (*mekk1/2/3*), defective in a set of three tandemly arranged MAP3Ks, was almost insensitive to l–Glu. However, the sensitivity of *mekk1/2/3* roots to inhibition by other amino acids reported to act as agonists of glutamate receptor-like (GLR) channels in Arabidopsis roots (Asn, Cys, Gly and Ser) was unaffected. The l–Glu sensitivity of the *mekk1/2/3* mutant was restored by transformation with a construct carrying the intact *MEKK1* gene. These results demonstrate that *MEKK*1 plays a key role in transducing the l–Glu signal that elicits large-scale changes in root architecture, and provide genetic evidence for the existence in plants of an l–Glu signalling pathway analogous to that found in animals.

## Introduction

The availability and distribution of nutrients in the soil have long been known to have a major influence on root architecture (Nobbe, 1862). The classic example is the localized proliferation of lateral roots (LRs) seen when the root systems of many plant species encounter a localized patch of nitrate (Robinson, 1994). However, although nitrate is the major source of nitrogen (N) for plants growing in fertile, aerobic soils, there is an increasing awareness of the significance to plants of amino acids and other organic N sources, particularly in the low-fertility soils of temperate regions (Schimel and Bennett, 2004; Rothstein, 2009). Consistent with the idea that plants should also have the ability to modify their root architecture to exploit organic N–rich patches, it has been found that the external presence of even low concentrations of l–Glu (<50 μm in some genotypes) can elicit major changes in root system architecture, resulting from the inhibition of primary root growth combined with increased LR proliferation behind the root apex (Walch-Liu *et al*., 2006). The requirement for direct contact between the primary root tip and l–Glu, and the specificity with which the roots responded to low concentrations of this amino acid, suggested that l–Glu was acting as an exogenous signal at the root tip (Walch-Liu *et al*., 2006). It was proposed that l–Glu could provide a chemical cue for the presence of localized sources of organic N in the soil, and that the subsequent change in root architecture could be an adaptive ‘foraging’ response that increases the precision of root placement within the organic N–rich patch (Walch-Liu *et al*., 2006; Forde and Lea, 2007). The use of amino acids as chemical foraging cues is a well-established phenomenon in a wide variety of motile organisms (Lux and Shi, 2004; Ide *et al*., 2006).

Interest in l–Glu as a possible signalling molecule in plants began with the discovery that Arabidopsis has a family of glutamate receptor-like (GLR) genes homologous to the mammalian ionotropic glutamate receptors (iGluRs; Lam *et al*., 1998). More recently, it has been reported that at least some members of the GLR family are able to act, like their mammalian counterparts, as amino acid-gated Ca^2+^ channels (Qi *et al*., 2006; Stephens *et al*., 2008; Michard *et al*., 2011; Vincill *et al*., 2012). In mammals, signal transduction downstream of iGluRs generally involves protein phosphorylation cascades (Wang *et al*., 2007); however, until now, it has been unclear how an external l–Glu signal is transduced to a downstream physiological or developmental response in plants.

The use of small molecules to probe gene function can overcome some of the limitations of conventional genetics, such as lethality, pleiotropic effects and redundancy of gene function, which are often a feature of genetic mutants (Toth and van der Hoorn, 2010). Here, we describe the results of a chemical genetics approach aimed at elucidating the molecular basis for the root's ability to sense and respond to external l–Glu. The development of the 96-well microphenotyping system described here allowed us to test >1600 bioactive small molecules, including 80 agonists and antagonists of mammalian iGluRs, metabotropic glutamate receptors (mGluRs) and γ–amino butyric acid receptors (GABARs), for their ability to alleviate the effect of l–Glu on root growth and branching. Although none of the previously characterized agonists or antagonists were active in this screen, we succeeded in identifying two groups of l–Glu antagonists from a collection of 1576 molecules known to be bioactive in *Saccharomyces cerevisiae* (yeast). Previous evidence that one of these l–Glu antagonists is active against an evolutionarily conserved MAP kinase kinase kinase (MAP3K) in yeast (Kitagawa *et al*., 2007) led to the demonstration that transduction of the l–Glu signal at the root tip involves *MEKK1*, a *MAP3K* gene previously implicated mainly in defence signalling (Ichimura *et al*., 2006; Nakagami *et al*., 2006; Suarez-Rodriguez *et al*., 2007; Rasmussen *et al*., 2012).

## Results

### Using a specially developed microphenotyping system in a targeted approach to screen for l–Glu antagonists

**Figure 1:**
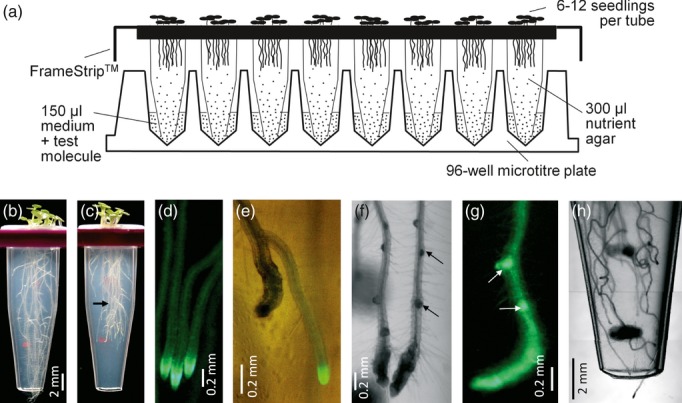
The 96-well microphenotyping method developed to screen for l–Glu agonists and antagonists by monitoring chemically induced changes in root growth and development. (a) Diagrammatic cross section of the device. Seedlings (GAL4-GFP line J2301) were grown in agar-filled microtubes (with between six and 12 seeds per tube, and with eight tubes per strip) that rested in the V–shaped wells of a microtitre plate. Test molecules were applied 2–3 days after germination by diffusion through the cut ends of the microtubes. l–Glu treatments, when applied, were initiated 2 days later (see Experimental Procedures). (b) Control (untreated) seedlings photographed 12 days after germination. (c) Seedlings (12 days old) that had been treated with l–Glu 5 days after germination (with a theoretical final concentration of 100 μm). The arrow indicates the position of the most advanced primary root tip at the time of treatment. (d) Fluorescence image of the untreated primary root tips of J2301 taken *in situ* and showing the normal pattern of GFP expression. (e) Micrograph of an l–Glu-treated root taken *in situ* using a combination of visible and fluorescent light, showing GFP expression in the lateral root (LR) tip and its absence in the distorted, growth-inhibited primary root tip. (f) Light micrograph of roots 9 days after treatment with 20 μm PHCCC showing an inhibited primary root with abnormal root tip morphology and stunted LRs (arrowed). (g) Fluorescence image showing pattern of GFP expression in a root treated with 4 μm PHCCC (8 days after treatment). Arrows indicate developmentally inhibited LRs. (h) Light micrograph of agravitropic roots 9 days after treatment with 20 μm SDZ 220–040.

A 96–well screening method was devised to enable the effects of large numbers of small molecules on root development to be analysed in detail (Figure 1a). The method was used to screen molecules both for their direct effects on root development and for their ability to block the effects caused by l–Glu. In the latter case, the test molecule was applied first, 2–3 days after germination, and the l–Glu was applied 2 days later, to allow time for a potential antagonist to exert its effect. Figure 1(b) shows untreated seedlings and Figure 1(c) shows seedlings treated with l–Glu only, illustrating how the distinctive l–Glu-elicited root phenotype can be readily observed in this system. An additional signature for the l–Glu effect was obtained using the J2301 enhancer trap line (Berger *et al*., 1998), which expresses GFP mainly in the root apex (Figure 1d). J2301 was found to respond to l–Glu treatment with a loss of GFP expression in the growth-inhibited primary root tip, but not in the still actively growing LR tips (Figure 1e).

Because of the known homologies between plant GLRs and the ancestrally related families of animal iGluRs, mGluRs and GABARs (Turano *et al*., 2001), we began with a targeted approach in which a set of 80 known agonists or antagonists of these mammalian receptors (Table S1) were screened for possible antagonists of the l–Glu response in roots. The pharmaceuticals were tested in duplicate at three concentrations (4, 20 and 100 μm), with and without the subsequent addition of l–Glu; however, none were found to alleviate the inhibitory effect of l–Glu, although a significant number on their own affected the root phenotype in different ways (Table S1). Two examples of this are shown in Figure 1. Root growth was strongly inhibited by *N*–phenyl-7-(hydroxyimino)cyclopropa[b]chromen-1a-carboxamide (PHCCC), a group–1 mGluR antagonist and a positive allosteric modulator of mGluR4 (Figure 1f). Although the PHCCC-induced thickening and curvature of the primary root tip was similar to that of an l–Glu-inhibited root, GFP fluorescence in this case was not lost (Figure 1g), and the LRs were highly stunted (Figure 1f,g). SDZ 220-040, a competitive antagonist of the mammalian NMDA receptor, induced a partially agravitropic pattern of root growth (Figure 1h).

### Screening for l–Glu antagonists amongst a collection of bioactive yeast molecules

**Figure 2:**
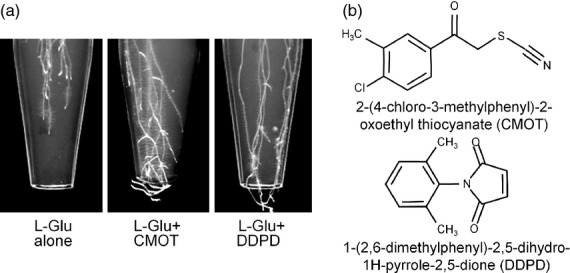
A chemical library screen identified two groups of molecules that act as l–Glu antagonists. (a) Using the microphenotyping system described in Figure 1, test molecules from the LATCA collection were applied 2 days after germination and l–Glu was applied 2 days later. The panel on the left shows roots treated with l–Glu alone and the other panels show the identification in the primary screen of two molecules (CMOT and DDPD) that overcame the effect of l–Glu on root growth and branching. (b) Chemical structures of CMOT and DDPD. These represent two separate groups of structurally related molecules identified as l–Glu antagonists in this screen.

The Library of Annotated Compounds for Arabidopsis (LATCA) collection includes 1576 inhibitors of yeast growth identified from a screen of 50 000 Maybridge compounds (http://cutlerlab.blogspot.com/2008/05/latca.html). Our micro-phenotyping system was used to screen this enriched set of bioactive molecules in duplicate for potential l–Glu antagonists. Figure 2(a) shows two examples from the primary screen where pre-treatment with a test molecule prevented the subsequent inhibition of primary root growth by l–Glu. These two molecules, 2–(4–chloro-3-methylphenyl)-2-oxoethyl thiocyanate (CMOT) and 1–(2,6–dimethylphenyl)-2,5-dihydro-1H-pyrrole-2,5-dione (DDPD), the structures of which are shown in Figure 2(b), belong to two distinct groups of structurally related compounds that were found to alleviate, or partially alleviate, the inhibitory effect of l–Glu. CMOT belongs to a group of active molecules that includes three other aromatic thiocyanates as well as a related molecule, 1–[2–(4–chlorophenyl)-2-oxoethyl]ethanedithioyl dicyanide (Table S2). The set of antagonists to which DDPD belongs are maleimides with a single aromatic group, 32 of which were present in the LATCA collection (Table S3). Of these, eight were identified as positives in the primary screen, but only DDPD has been investigated further.

### Testing the specificity of the l–Glu antagonists

**Figure 3:**
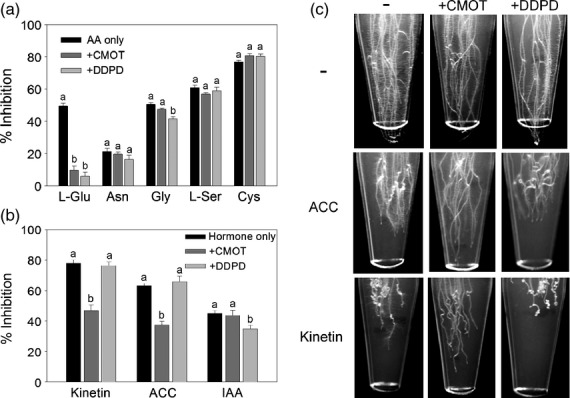
Effect of l–Glu antagonists on root growth inhibition by other amino acids and plant hormones. Seedlings were cultivated in the microphenotyping system described in Figure 1. (a) Antagonists (with a theoretical final concentration of 8.3 μm) were added to the microtitre wells 3 days after germination, and amino acids were added 2 days later to give theoretical final concentrations of 1 mm (Asn and Gly) and 0.5 mm (Cys and Ser). Root growth over the following 3 days was measured and the percentage inhibition was calculated by comparison with the corresponding (no amino acid) treatment (±SEM; *n* = 8). Different letters indicate statistically significant differences between groups (*P* < 0.05). (b) As for (a), except that the antagonist and hormone treatments were each given 1 day earlier and growth was measured over the 4–day period after hormone treatment (±SEM; *n* = 8). The theoretical final concentrations were: 330 nm (ACC and kinetin) and 33 nm (IAA). (c) Images showing the effect of CMOT and DDPD on the growth and morphology of roots treated with ACC or kinetin. Treatments were as described for (b), except that antagonist and hormone treatments were each given 1 day later. Images were captured 6 days after hormone treatments.

We investigated whether the antagonistic activities of CMOT and DDPD are specific to l–Glu or extend to other amino acids or hormones that inhibit root growth. The four amino acids tested (Gly, Asn, Cys and Ser) were chosen for their reported roles as agonists or regulators of GLR glutamate receptors in Arabidopsis (Dubos *et al*., 2003; Qi *et al*., 2006; Stephens *et al*., 2008; Michard *et al*., 2011; Vincill *et al*., 2012), and for their ability to inhibit root growth in the concentration range 0.5–1 mm, as established in preliminary experiments. The results in Figure 3(a) confirm the ability of both CMOT and DDPD to antagonize the inhibitory effect of l–Glu and demonstrate their specificity for l–Glu. It was only in the case of Gly and the DDPD treatment that a minor antagonistic effect was observed; neither CMOT nor DDPD suppressed the inhibitory effect of the other amino acids.

We found that CMOT was able to significantly reduce the inhibitory effect of both the ethylene precursor aminocyclopropane-1-carboxylic acid (ACC) and kinetin (Figure 3b,c), but had no effect on the inhibitory effect of indole acetic acid (IAA; Figure 3b). In contrast, DDPD had a small counteractive effect on inhibition by IAA, but had no effect on inhibition by either ACC or kinetin (Figure 3b,c). Note that, as seen in the top panels of Figure 3(c), neither CMOT nor DDPD at the concentrations used in these experiments had major effects on primary root elongation when present on their own.

### Investigating the role of MAP kinases in the l–Glu response

Previously, in a screen of approximately 8000 small molecules, CMOT was identified as being able to specifically block the activity of the Ste11 MAP3K in yeast (Kitagawa *et al*., 2007). The A1 subgroup of Ste11-related MAP3Ks in Arabidopsis includes the *MEKK1* gene, which can complement a yeast Ste11 mutant (Mizoguchi *et al*., 1996), and the closely related *MEKK2*, *MEKK3* and *MEKK4* genes (Ichimura *et al*., 2002).

**Figure 4:**
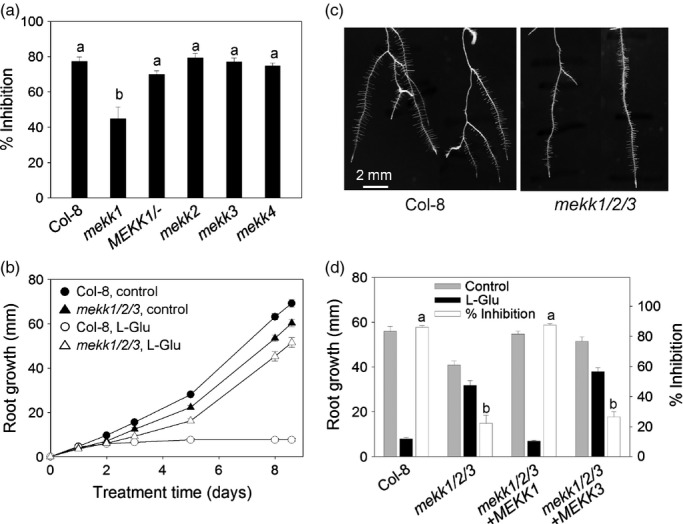
Effect of mutations in *MEKK1*-related genes on root sensitivity to l–Glu. (a) Seedlings (5 days old) of parental (Col–8) and mutant lines were transferred to vertical agar plates containing 0.5 mm Gln as a source of N, and either 2 mm KCl (control) or 2 mm l–Glu. For each line the percentage inhibition of primary root growth by l–Glu over the subsequent 6 days was calculated by comparison with the control treatment (±SEM; *n* = 8–28). The *mekk1* mutant was only available as segregating progeny from a *MEKK1 mekk1*–*1* heterozygote, but the dwarf shoot phenotype of homozygous *mekk1–1* seedlings (Suarez-Rodriguez *et al*., 2007) allowed the l–Glu sensitivity to be scored separately from *MEKK1*/–seedlings. (b) Time course of primary root growth of Col–8 and *mekk1/2/3* seedlings after transfer to control or l–Glu plates. (c) Images taken after 5 days of growth on 2 mm l–Glu, showing contrasting patterns of lateral root (LR) proliferation in the apical regions of primary roots of Col–8 and *mekk1/2/3* seedlings. (d) Primary root growth of the *mekk1/2/3* mutant and *mekk1/2/3* rescue lines transformed with the *MEKK1* gene (*mekk1/2/3 + MEKK1*) or the *MEKK3* gene (*mekk1/2/3 + MEKK3*) after 8 days on medium, with or without 0.5 mm l–Glu (±SEM; *n* = 9–12). The percentage inhibition by l–Glu has also been plotted for each line. Different letters indicate statistically significant differences between groups (*P* < 0.05).

To investigate whether *MEKK1* or the other *MEKK1*-related genes are involved in l–Glu signalling, we began by testing the l–Glu sensitivity of root growth in single knock-out mutants of *MEKK1* and the other three A1 subgroup members. Because the *mekk1–1* mutant is infertile (Ichimura *et al*., 2006; Nakagami *et al*., 2006; Suarez-Rodriguez *et al*., 2007), it was necessary to analyse the segregating progeny of a *MEKK1 mekk1–1* heterozygote, the distinctive dwarf phenotype of the homozygous mutant, making it possible to score root growth of *mekk1–1 mekk1–1* and *MEKK1/–* seedlings separately. Roots of *mekk1–1 mekk1–1* seedlings were slower growing than the parental line in the absence of l–Glu, but were much less sensitive to l–Glu, whereas *MEKK1/–* roots grew normally in the absence of l–Glu and were inhibited to the same degree as the wild type (Figure 4a). By contrast, the *mekk2* (*summ1–1)*, *mekk3* and *mekk4* single knock-out mutants were unaltered in their l–Glu sensitivity (Figure 4a).

The pleiotropic phenotype of the *mekk1–1* mutant (Ichimura *et al*., 2006; Nakagami *et al*., 2006; Suarez-Rodriguez *et al*., 2007) complicates our ability to interpret its reduced sensitivity to l–Glu. It has recently been demonstrated that the dwarf and autoimmune phenotypes of *mekk1–1* can be suppressed by inactivating the *MEKK2* gene (Kong *et al*., 2012), and consistent with this it has recently been found that a deletion mutation that disrupts the entire *MEKK1–MEKK2–MEKK3* gene cluster (*mekk1/2/3*) is phenotypically similar to the wild type (Su *et al.,* 2013). Use of the *mekk1/2/3* mutant therefore allowed us to study the effect on l–Glu sensitivity of loss of *MEKK1* function without the complication of the pleiotropic phenotype of the *mekk1* single mutant. The triple mutant was found to be almost insensitive to l–Glu, with primary root growth continuing for at least 8 days at only a slightly reduced rate in the presence of 2 mm l–Glu compared with the control (Figure 4b). Furthermore, the dramatic effect on root architecture elicited by l–Glu in the wild type (Walch-Liu *et al*., 2006) was absent in the *mekk1/2/3* mutant (Figure 4c), as quantified by expressing the total LR length per unit primary root length in the root zone developing after treatment (Figure S1).

To establish whether the absence of *MEKK1* was responsible for the l–Glu insensitivity displayed by the *mekk1/2/3* plants, we used a transgenic rescue line (*mekk1/2/3 + MEKK1*) in which the *mekk1/2/3* mutant carried the wild-type *MEKK1* gene driven by its native promoter (Su *et al.,* unpublished data). Both LR and primary root growth, which are diminished in *mekk1/2/3*, are restored to wild-type levels in this rescue line (data not shown). Figure 4(d) shows that l–Glu sensitivity was also fully restored in *mekk1/2/3 + MEKK1*, whereas l–Glu sensitivity in another rescue line (*mekk1/2/3 + MEKK3*), carrying a wild-type copy of *MEKK3* (Su *et al*., unpublished data), was very similar to *mekk1/2/3* itself (Figure 4d).

The transgenic line *mekk1 + K361M* is a *mekk1–1* mutant expressing a mutant version of MEKK1 in which Lys361, a conserved residue essential for normal kinase activity, has been substituted by Met (Suarez-Rodriguez *et al*., 2007). This line, which is fertile and grows normally, was previously used to provide evidence that the role of MEKK1 in the response of the plant to the flagellin elicitor peptide flg22 is independent of its full protein kinase activity (Suarez-Rodriguez *et al*., 2007). We found that root growth in *mekk1 + K361M* was as sensitive to l–Glu as in the wild type (Figure S2), indicating that the kinase activity of MEKK1 is similarly not required for l–Glu signalling.

### Sensitivity of the *mekk1/2/3* triple mutant to other inhibitors of root growth

**Figure 5:**
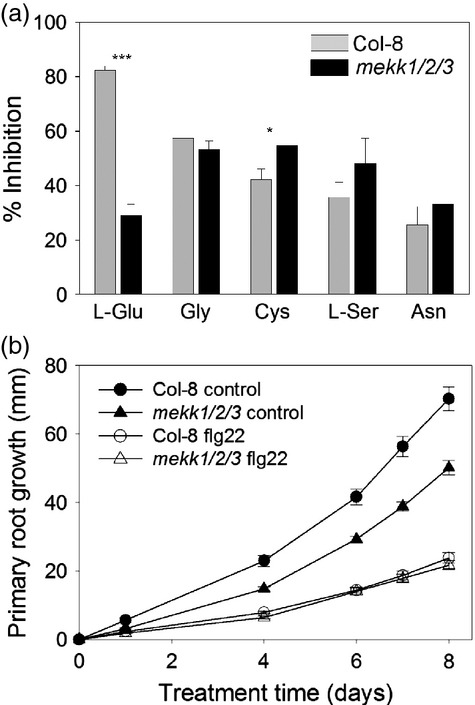
Sensitivity of the *mekk1/2/3* mutant to other amino acids. (a) Seedlings (5 days old) of Col–8 and *mekk1/2/3* were transferred to plates containing either 0.5 mm Gln alone (control) or 0.5 mm Gln plus 0.5 mm l–Glu, 2 mm Gly, 0.5 mm Cys, 1 mm Ser or 2 mm Asn. The percentage inhibition of primary root growth by each amino acid was determined by measuring primary root growth over the following 8 days and comparing it with the control. Asterisks indicate where the difference between the parental line and the mutant is statistically significant (Student's *t*–test: *P < 0.05; ***P < 0.001). (b) Time course of primary root growth of Col–8 and *mekk1/2/3* seedlings after transfer to control plates or plates containing 1 μm flg22.

The l–Glu specificity of the *mekk1/2/3* mutant phenotype was investigated by testing the sensitivity of its roots to inhibition by the other four previously selected amino acids. Figure 5(a) shows that the triple mutant was at least as sensitive to Cys, Gly, l–Ser and Asn as the wild type. Note that the slightly increased sensitivity to Cys seen here was not reproduced in other experiments.

One of roles of MEKK1 is as part of a MAP kinase cascade, downstream of the flagellin receptor FLS2, which detects the presence of pathogen-associated molecular patterns (PAMPS) such as the flagellin peptide flg22 (Ichimura *et al*., 2006; Nakagami *et al*., 2006; Suarez-Rodriguez *et al*., 2007; Gao *et al*., 2008). As one of the plant responses to flg22 is the strong inhibition of root growth (Gomez-Gomez *et al*., 1999), we considered the possibility that l–Glu and flg22 could act on root growth through the same MEKK1-mediated pathway; however, as shown by the growth curves in Figure 5(b), *mekk1/2/3* roots were almost as sensitive to inhibition by flg22 as wild type roots (56 and 66% inhibition, respectively), indicating that flg22 affects root growth largely through a MEKK1-independent pathway.

## Discussion

Pharmacological approaches have been fundamental to the progress made in understanding l–Glu signalling pathways in mammals, and a wide range of small molecules have been developed that act as agonists or antagonists of mammalian iGluRs and mGluRs (Watkins and Jane, 2006). Although some of these are able to disrupt the electrophysiological or Ca^2+^ flux responses to l–Glu in plants (Dubos *et al*., 2003; Sivaguru *et al*., 2003; Meyerhoff *et al*., 2005; Teardo *et al*., 2010; Kwaaitaal *et al*., 2011), their precise targets have not yet been verified. Neither of the iGluR antagonists most commonly used in plants (AP–5 and DNQX) was able to suppress the inhibitory effect of l–Glu on root growth (Walch-Liu *et al*., 2006; Walch-Liu and Forde, 2007). Here, we expanded on this work by surveying a collection of 80 agonists and antagonists of mammalian iGluRs, mGluRs and GABARs. Whereas a significant proportion (17.5%) were phytoactive (as demonstrated by their ability to affect root growth, LR development, root hair production and/or root gravitropism), none were fully able to replicate the l–Glu effect in a way that suggested they were acting as l–Glu agonists, and nor did any antagonize the response to l–Glu. This might suggest that members of the plant GLR family are not involved in perception of the l–Glu signal that leads to changes in root development. However, it is also possible that the particular GLR(s) involved in this response are sufficiently divergent from their mammalian counterparts to not be effective targets of the pharmaceuticals, or that the pharmaceuticals that do interact with the multiple GLRs expressed in roots (Chiu *et al*., 2002) are amongst the group that induce pleiotropic effects on root development (Figure 1; Table S1), and that these mask the phenotypes for which we were screening.

By extending our screen to a collection of >1500 small molecules that are bioactive in yeast, we identified two groups of compounds that were able to interfere with the effect of l–Glu on root development. Four of the active molecules were aromatic thiocyanates (of which CMOT was the representative), whereas the other group comprised a series of aromatic maleimides (of which DDPD was the representative; Figure 2; Tables S1 and S2). When tested for their ability to antagonize the inhibitory effect on root growth of three plant hormones, CMOT and DDPD behaved in contrasting ways, suggesting that they have different targets (Figure 3b,c). A report that cytokinin inhibits root elongation in part through ethylene signalling (Ruzicka *et al*., 2009) suggests a way that CMOT could partially overcome growth inhibition by both ACC and kinetin by targeting the ethylene signalling pathway.

Evidence that CMOT (previously BTB03006) targets the Ste11 MAP3K in yeast (Kitagawa *et al*., 2007) led us to investigate the role of Ste11-related MAP3Ks in l–Glu signalling in roots. MEKK1, which plays a signal transduction role in diverse biotic and abiotic signalling pathways, is the most studied member of the A1 subgroup of Ste11-related MAP kinases in Arabidopsis (Rodriguez *et al*., 2010). It belongs to the same tandem gene cluster as *MEKK2* and *MEKK3*, with *MEKK4* being a more divergent member of the same subgroup (Champion *et al*., 2004). Root growth in a *mekk1* mutant was found to be significantly less sensitive to l–Glu than the wild type, whereas *mekk2*, *mekk3* and *mekk4* knock-out mutants were unaltered in their l–Glu sensitivity (Figure 4).

Interpreting the l–Glu sensitivity of the *mekk1* mutant is complicated by its severe dwarf and autoimmune phenotypes. To study the loss of *MEKK1* function in a phenotypically normal background, we were able to take advantage of a recently isolated deletion mutant that disrupts all three of the tandemly arranged *MEKK1*, *MEKK2* and *MEKK3* genes (Su *et al*. unpublished data). As a result of the loss of *MEKK2* the *mekk1/2/3* plants are phenotypically normal (Kong *et al*., 2012; Su *et al*. unpublished data), but were almost insensitive to l–Glu (Figure 4). Furthermore, the genetic rescue of *mekk1/2/3* with the intact *MEKK1* gene was sufficient to fully restore wild-type levels of l–Glu sensitivity, demonstrating that *MEKK1* is required for the normal functioning of the l–Glu signalling pathway. Previous evidence that the *MEKK1* promoter directs expression mainly to the primary root tip and lateral root primordia (Su *et al*., 2007) is consistent with the proposed signalling role of MEKK1 in the response of the root to external l–Glu.

Previous studies with a *mekk1* + *K361M* transgenic line, which expresses a kinase-defective version of *MEKK1* in the *mekk1–1* background, provided evidence that its kinase activity is not required for MEKK1 to function as a component of the pathogen response pathway (Suarez-Rodriguez *et al*., 2007). The wild-type levels of l–Glu sensitivity we observed in *mekk1* + *K361M* (Figure S2) suggest that the kinase activity of MEKK1 is similarly not required for l–Glu signalling. To explain the functionality of kinase-impaired MEKK1 in the pathogen response, it was previously proposed that the MEKK1 protein could serve a structural role as a scaffold for other MAP kinases in the signalling cascade (Suarez-Rodriguez *et al*., 2007). Alternatively, there is evidence that MEKK1 is able to function directly as a DNA binding protein, having been found to interact with the promoter of the gene for the *WRKY53* transcription factor, thereby bypassing the usual downstream MAP kinase cascade (Miao *et al*., 2007).

It is well established that treating Arabidopsis roots with l–Glu (as well as a number of other amino acids) elicits rapid changes in membrane potential and increased Ca^2+^ fluxes (Demidchik *et al*., 2004), and there is evidence that specific members of the GLR family are required for this response (Qi *et al*., 2006; Stephens *et al*., 2008; Michard *et al*., 2011; Vincill *et al*., 2012). It was recently reported that the treatment of Arabidopsis seedlings with l–Glu led to the slight activation of a group of MAP kinases, a response that was sensitive to an iGluR antagonist (Kwaaitaal *et al*., 2011). In mammals, MAP kinase pathways play an important signalling role in the iGluR-mediated response to l–Glu (Mao *et al*., 2004; Haddad, 2005; Wang *et al*., 2007); however, the major pathways appear to involve Raf-type MAP3Ks rather than the mammalian Ste11-related MAP3Ks that are more closely related to Arabidopsis MEKK1 (Fukunaga and Miyamoto, 1998; Mao *et al*., 2004; Garcia *et al*., 2008). Thus the intriguing possibility that there is evolutionary conservation of l–Glu signalling pathways between plants and animals cannot be confirmed.

We have shown that when applied at relatively high concentrations (in the range of 0.5–2 mm), a number of amino acids (Asn, Cys, Gly and Ser) are able to partially inhibit root growth (Figures 3a and 5), although none elicited the dramatic effects on growth and root architecture previously seen with l–Glu (Walch-Liu *et al*., 2006). Each of these amino acids has been implicated (along with l–Glu) as a ligand for either one or both of the GLR3.3 and GLR3.4 receptor channel subunits in Arabidopsis roots (Qi *et al*., 2006; Stephens *et al*., 2008; Vincill *et al*., 2012). However, the finding that CMOT has no alleviating effect on growth inhibition by Asn, Cys, Gly or Ser (Figure 3a), and that the *mekk1/2/3* mutant remains sensitive to all four amino acids (Figure 5), indicates that these amino acids affect root growth via mechanisms that are distinct from the l–Glu signalling pathway identified here.

We have not pinpointed the exact targets of either of the two groups of l–Glu antagonists identified in this study. In yeast, the genetic evidence indicated that CMOT acts either on Ste11 itself or on another protein required for activation of Ste11 (Kitagawa *et al*., 2007). The l–Glu-insensitive phenotype of the *mekk1/2/3* mutant makes it likely that CMOT targets either this subgroup of Ste11-related MAP kinases or another regulatory protein required for their activation.

The present work provides a clear example of the value of a chemical genetics approach, as the infertility and dwarf phenotype of a homozygous *mekk1* mutation (Ichimura *et al*., 2006; Nakagami *et al*., 2006; Suarez-Rodriguez *et al*., 2007) would have precluded the identification of *MEKK1* as a component of the l–Glu signalling pathway in a conventional forward genetics screen. Furthermore, it is worth noting that the process described here would have been a much greater challenge had it not been for the availability of a highly enriched collection of bioactive molecules identified from a yeast chemical genetics screen (http://cutlerlab.blogspot.com/2008/05/latca.html), and the previous identification of Ste11 as a target for one of these molecules in yeast (Kitagawa *et al*., 2007).

## Experimental Procedures

### Plant materials and small molecules for screening

The wild-type *Arabidopsis thaliana* L. accession Col–8 and the green fluorescent protein (GFP) enhancer trap line J2301 (20) were obtained from the European Arabidopsis Stock Centre (catalogue nos N60000 and N9173, respectively; http://arabidopsis.info). The *mekk1–1* T–DNA knock-out mutant and the *mekk1* + *K361M* transgenic line were described previously (Suarez-Rodriguez *et al*., 2007; Bush and Krysan, 2010). The T–DNA insertion lines for *MEKK3* (At4 g08470) and *MEKK4* (At4 g12020) were SALK_093491 and SALK_097632, respectively (Alonso *et al*., 2003). The *summ1–1* mutant, in which *mekk2* is disrupted by an early stop codon, was the gift of Prof. Yuelin Zhang (University of British Columbia). The *mekk1/2/3* mutant, described in detail elsewhere (Su *et al*. unpublished data) is a triple null mutant that has lost a approximately 20–kb segment of chromosome 4, extending from the middle of *MEKK1* to the middle of *MEKK3*. The *MEKK1* rescue construct carried the wild-type *MEKK1* locus under the transcriptional control of its native promoter (Suarez-Rodriguez *et al*., 2007), and was moved into the *mekk1/2/3* mutant background by genetic crossing (to create *mekk1/2/3 + MEKK1*). The transgenic event carrying the *MEKK1* rescue construct that was used for this cross was previously shown to be able to fully rescue the dwarf *mekk1* mutant phenotype (Suarez-Rodriguez *et al*., 2007). The *MEKK3* rescue construct carried the *MEKK3* locus under the transcriptional control of its native promoter (Su *et al*. unpublished data), and was introduced into *mekk1/2/3* by *Agrobacterium* transformation (to create *mekk1/2/3 + MEKK3*). Both the resulting transgenic lines used here express the rescue construct at levels similar to the endogenous gene in the wild type (Su *et al*. unpublished data). The 80 agonists and antagonists of mammalian glutamate and GABA receptors were from Tocris Bioscience (http://www.tocris.com), and molecules used for follow-up studies were from Thermo Fisher Scientific (http://www.thermofisher.com).

### Microphenotyping system

Strips of eight PCR tubes (FrameStrip™; 4titude, http://www.4ti.co.uk), supported in 96-well PCR boxes (Thermo Fisher Scientific), were filled with approximately 300 μl of nutrient agar (1/20 Gamborg's B5 medium, 20 μm NH_4_NO_3_, 0.8% agar, 0.5% sucrose). Sterilized seed of GFP enhancer trap line J2301 was sown onto the agar surface (with between six and 12 seeds per tube), stratified in the dark at 3°C for 2–4 days and transferred to the growth room at 22°C with a 16–h light/8–h dark photoperiod at a light intensity of 38 μmol m^−2^ s^−1^. The PCR boxes were kept in propagators lined with moistened absorbent paper towel to maintain humidity. After 2–3 days the bottom approximately 2 mm of each tube was excised using a guillotine and the strips transferred to 96–well microtitre plates containing 150 μl of 1/50 B5 medium. Compounds from the LATCA collection (2.5 mm in DMSO) were added as 1.5–μl aliquots to each well (final theoretical concentration 8.3 μm, assuming equilibration within the approximately 450–μl assay volume). Tocris compounds (10 mm in DMSO) were applied at three theoretical final concentrations: 100, 20 and 4 μm. After a further 2 days, to allow time for the small molecules to diffuse into the root zone, 45 nmol of l–Glu + 45 nmol Gln (as an additional N source) were added to each well. In treatments where l–Glu was omitted, 68 nmol Gln was added. For high-throughput screening the roots were observed after a minimum of a further 3–4 days by removing the strips individually and scoring visually for presence or absence of the distinctive l–Glu-elicited root phenotype (Figure 1e). Fluorescence and light microscopy of roots *in situ* was performed using a Leica MZFLIII stereomicroscope (Leica, http://www.leica-microsystems.com). A Canon 600D camera with a 60–mm f/2.8 lens was used for lower power imaging (Canon, http://www.canon.com). To measure root growth rates, the position of the tip of the most advanced primary root was marked at intervals on the side of each tube, images were captured using a Canonscan 4200 flat-bed scanner and analysed using optimas image analysis software (MediaCybernetics, http://www.mediacy.com). This microphenotyping system is the subject of a UK patent application (no. GB1218089.9).

### Vertical agar plate culture

Seedlings (5–6 days old) were tested for sensitivity to l–Glu or other amino acids, as previously described (Walch-Liu and Forde, 2008). l–Glu was supplied as the potassium salt, and all treatment and control (KCl) plates contained 0.5 mm Gln as a source of N.

### Statistical analysis

One-way anova analyses with Dunnett's *post-hoc* test were performed using spss 19 (IBM, http://www.ibm.com).

## SUPPORTING INFORMATION

**Figure S1.** The effect of the *mekk1/2/3* triple mutation on l–Glu elicited changes in root architecture.

**Figure S2.** Effect of a kinase-impaired *MEKK1* mutant on the l–Glu sensitivity of root growth.

**Table S1.** Pharmaceuticals screened for their ability to act as l–Glu agonists and antagonists.

**Table S2.** Molecules related to CMOT in the LATCA collection and their activity as l–Glu antagonists in the microphenotyping assay.

**Table S3.** Molecules related to DDPD that showed activity as l–Glu antagonists in the microphenotyping assay.
