# Supplementary figures and images for: Glutamate signalling via a MEKK1 kinase-dependent pathway induces changes in Arabidopsis root architecture
Source: Plant J. 2013 Apr 10;75(1):1–10. doi: 10.1111/tpj.12201 (PMC3739925; doi:10.1111/tpj.12201)

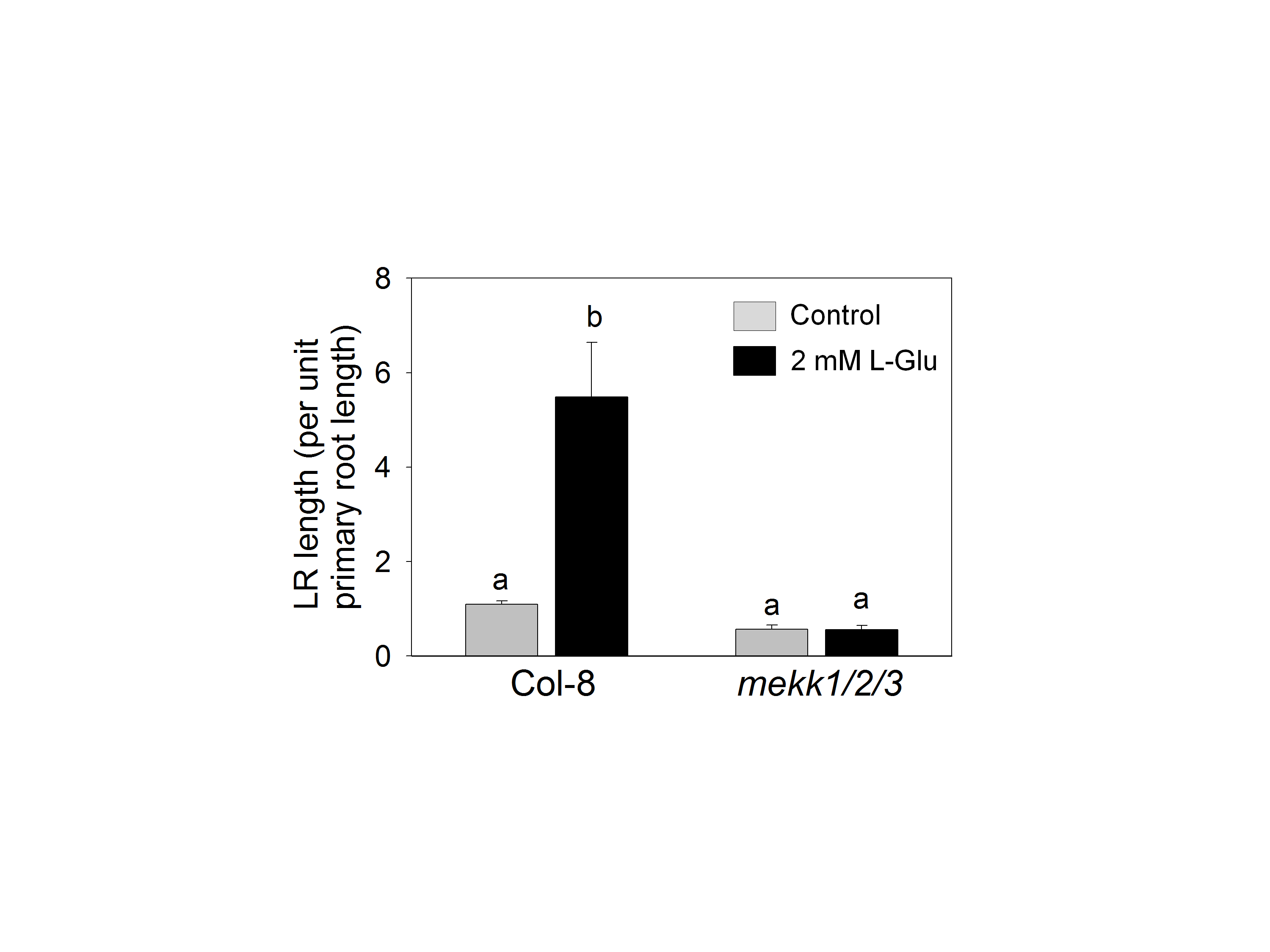

Supplement: Supplementary file 1 [file tpj0075-0001-SD1.tif]

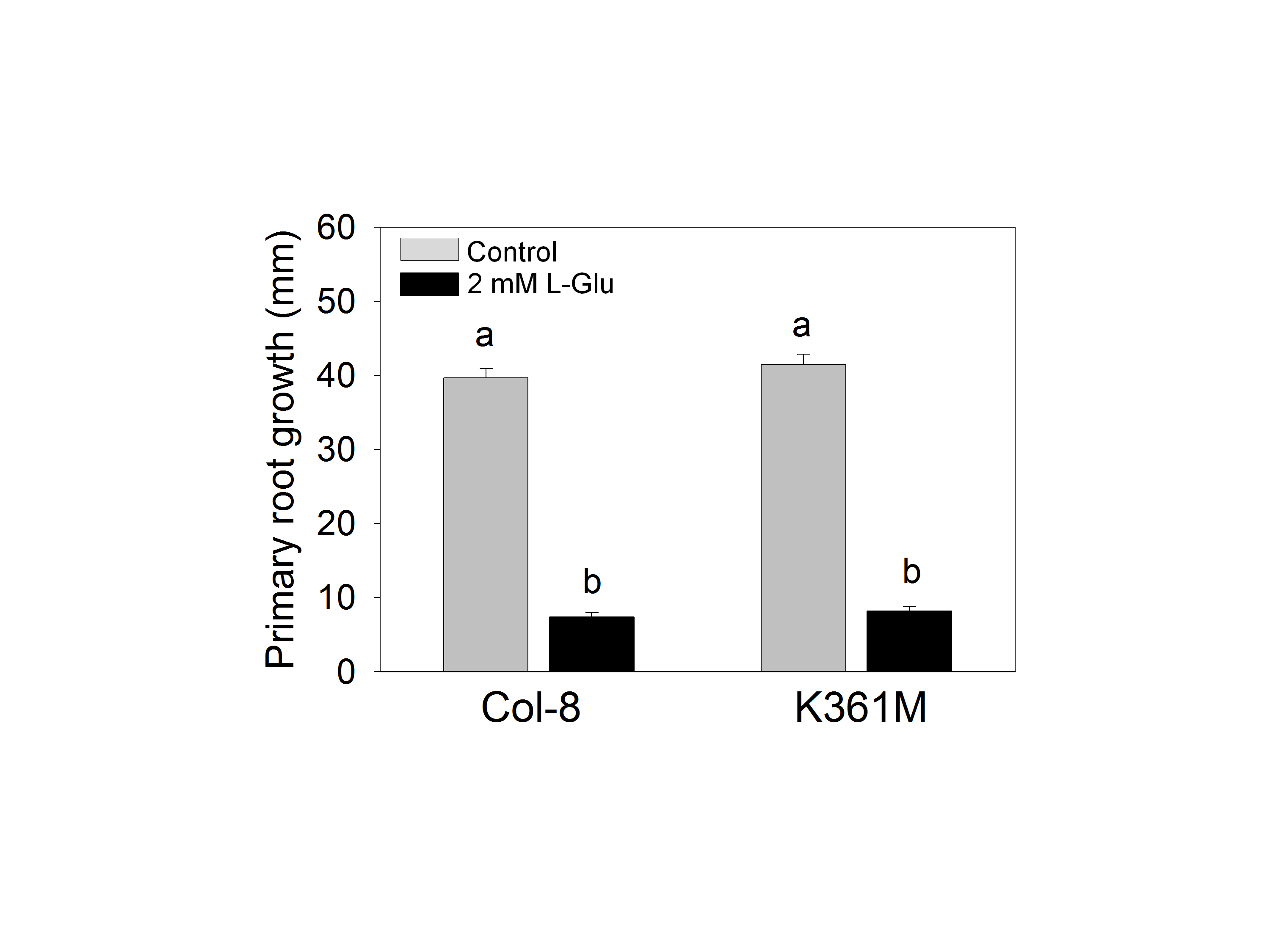

Supplement: Supplementary file 2 [file tpj0075-0001-SD2.tif]
