# Supplementary material for: Glutamate signalling via a MEKK1 kinase-dependent pathway induces changes in Arabidopsis root architecture
Source: Plant J. 2013 Apr 10;75(1):1–10. doi: 10.1111/tpj.12201 (PMC3739925; doi:10.1111/tpj.12201)
Supplement: Supplementary file 4 [file tpj0075-0001-SD4.docx]

**Table S2. Molecules related to CMOT in the LATCA collection and their activity as L-Glu antagonists in the micro-phenotyping assay**

| **Structure** | **LATCA referenceno.** | **Chemical name** | **Maybridge catalog no.** | **Antagonist** |
| --- | --- | --- | --- | --- |
| 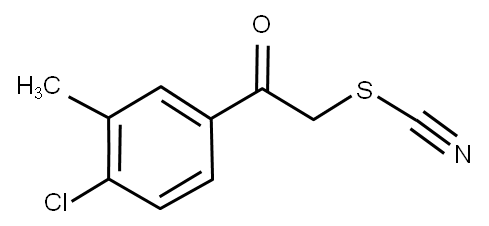 | LAT27G06 | 2-(4-chloro-3-methylphenyl)-2-oxoethyl thiocyanate (CMOT) | BTB03006 | Yes (+++) |
| 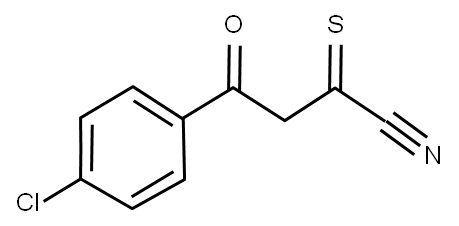 | LAT27G11 | 1-[2-(4-chlorophenyl)-2-oxoethyl]ethanedithioyl dicyanide | BTB06938 | Yes (++) |
| 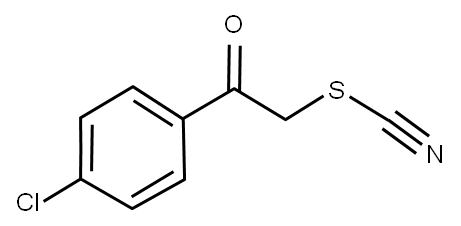 | LAT31H08 | 2-(4-chlorophenyl)-2-oxoethyl thiocyanate | KM04778 | Yes (+) |
| 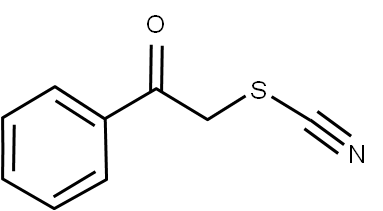 | LAT40C11 | 2-oxo-2-phenylethyl thiocyanate | DP00881 | Yes (+) |
| 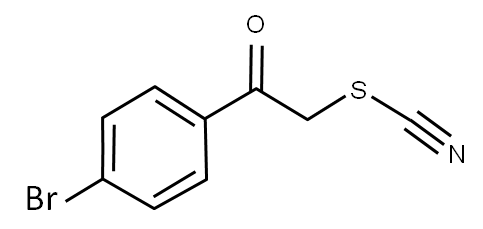 | LAT29B07 | 2-(4-bromophenyl)-2-oxoethyl thiocyanate | DP01545 | Yes (+) |
|  | LAT32F07 | 1-(4-chlorophenyl)-2-(3,4-dihydroisoquinolin-1-ylsulfanyl)ethan-1-one hydrobromide | MWP00441 | No |
