# Supplementary material for: Glutamate signalling via a MEKK1 kinase-dependent pathway induces changes in Arabidopsis root architecture
Source: Plant J. 2013 Apr 10;75(1):1–10. doi: 10.1111/tpj.12201 (PMC3739925; doi:10.1111/tpj.12201)
Supplement: Supplementary file 6 [file tpj0075-0001-SD6.docx]

**Table S1.** Pharmaceuticals screened for their ability to act as L-Glu agonists and antagonists

**Table S2.** Molecules related to CMOT in the LATCA collection and their activity as L-Glu antagonists in the micro-phenotyping assay

**Table S3.** Molecules in the LATCA collection related to DDPD that showed activity as L-Glu antagonists in the micro-phenotyping assay

**Figure S1**. Effect of the *mekk1/2/3* triple mutation on L-Glu elicited changes in root architecture. Five-d-old seedlings of Col-8 and *mekk1/2/3* were transferred to control plates or plates containing 2 mM L-Glu. Measurements were made on images taken after 8 d of treatment. The total length of LRs in the region of the primary root that developed after the start of treatment is expressed per unit length of that part of the primary root (±SEM; n=12-14). Different letters indicate statistically significant differences between groups (*P*<0.05).

**Figure S2.** Effect of a kinase-impaired *MEKK1* mutant on L-Glu sensitivity of root growth. Five-d-old seedlings of Col-8 and the *mekk*1+*K361M* line (Suarez-Rodriguez *et al*., 2007) were transferred to control plates or plates containing 2 mM L-Glu. Measurements were made on images taken after 6 d of treatment (±SEM; n=12-14). Different letters indicate statistically significant differences between groups (*P*<0.05).
